# GATA3 Expression Is Decreased in Psoriasis and during Epidermal Regeneration; Induction by Narrow-Band UVB and IL-4

**DOI:** 10.1371/journal.pone.0019806

**Published:** 2011-05-17

**Authors:** Emőke Rácz, Dorota Kurek, Marius Kant, Ewout M. Baerveldt, Edwin Florencia, Sabine Mourits, Dick de Ridder, Jon D. Laman, Leslie van der Fits, Errol P. Prens

**Affiliations:** 1 Department of Dermatology, Erasmus University Medical Center, Rotterdam, The Netherlands; 2 Department of Immunology, Erasmus University Medical Center, Rotterdam, The Netherlands; 3 Department of Cell Biology, Erasmus University Medical Center, Rotterdam, The Netherlands; 4 Information and Communication Theory Group, Faculty of Electrical Engineering, Mathematics and Computer Science, Delft University of Technology, Delft, The Netherlands; Institut Jacques Monod, France

## Abstract

Psoriasis is characterized by hyperproliferation of keratinocytes and by infiltration of activated Th1 and Th17 cells in the (epi)dermis. By expression microarray, we previously found the GATA3 transcription factor significantly downregulated in lesional psoriatic skin. Since GATA3 serves as a key switch in both epidermal and T helper cell differentiation, we investigated its function in psoriasis. Because psoriatic skin inflammation shares many characteristics of epidermal regeneration during wound healing, we also studied GATA3 expression under such conditions.

Psoriatic lesional skin showed decreased GATA3 mRNA and protein expression compared to non-lesional skin. GATA3 expression was also markedly decreased in inflamed skin of mice with a psoriasiform dermatitis induced with imiquimod. Tape-stripping of non-lesional skin of patients with psoriasis, a standardized psoriasis-triggering and skin regeneration-inducing technique, reduced the expression of GATA3. In wounded skin of mice, low GATA3 mRNA and protein expression was detected. Taken together, GATA3 expression is downregulated under regenerative and inflammatory hyperproliferative skin conditions. GATA3 expression could be re-induced by successful narrow-band UVB treatment of both human psoriasis and imiquimod-induced psoriasiform dermatitis in mice. The prototypic Th2 cytokine IL-4 was the only cytokine capable of inducing GATA3 in skin explants from healthy donors. Based on these findings we argue that GATA3 serves as a key regulator in psoriatic inflammation, keratinocyte hyperproliferation and skin barrier dysfunction.

## Introduction

Psoriasis is a very common chronic inflammatory skin disease characterized by sharply demarcated, thick, red, scaly plaques. Histologically it is characterized by epidermal acanthosis, papillomatosis and parakeratosis, infiltrating leukocytes and neutrophils in the epidermis and dermis, and neoangiogenesis.

In psoriasis, altered keratinocyte differentiation is characterized by downregulation of late keratinocyte differentiation markers and, upregulation of early differentiation markers [1], accompanied by an increase in the pool of proliferating keratinocytes. The alterations in keratinocyte proliferation and differentiation lead to impairment of the skin barrier function, and this barrier impairment correlates with the severity of the disease [2].

The factors controlling keratinocyte hyperproliferation and the disturbed keratinocyte differentiation in psoriasis remain incompletely understood. Hyperproliferation in psoriasis and proliferation in cancers share many characteristics, such as the induction of similar oncogenes and transcription factors [3]. Previous microarray studies assessing altered biological pathways in psoriasis consistently showed that mRNA encoding the transcription factor GATA3 was significantly downregulated in lesional psoriatic keratinocytes, and was re-induced by successful therapy [4], [5].

GATA3 is a transcription factor with two zinc finger motifs that binds to a six-nucleotide consensus sequence (A/T)GATA(A/G) [6]. In the skin, GATA3 is expressed in the epidermis and in the inner root sheath of the hair follicle [7] where it serves as a regulator of inner root cell lineage formation of the hair follicle, postnatal hair growth and maintenance [8], [9]. GATA3 is essential for correct formation of the epidermal barrier, in the regulation of epidermal differentiation and desquamation via activation of kallikrein 1 [10], [11]. In mice, complete GATA3 deficiency is incompatible with life. In the skin of epidermis-specific GATA3-deficient mice, production of antimicrobial peptides, such as β-defensins and S100A proteins is upregulated [9], [10]. In addition, GATA3 is abundantly expressed in the developing nervous system, inner ear, the eye, skin, mammary glands, embryonic kidney and thymus [8], [9], [12], [13], [14]. In cells of hemopoietic origin, GATA3 expression is confined to the T, NK and NKT cell lineages [15].

During lymphoid cell development, GATA3 is involved in T cell commitment [16]. Functional T helper cell subset differentiation into Th2 is induced by GATA3 via a STAT6-dependent route [17], [18], [19], [20]. Polymorphisms in the Th2 cytokine genes IL-4 and IL-13 have been reported in psoriasis [21], indicating that not only a dominant Th1/Th17 axis but also a defective Th2 axis can affect the disease.

As psoriasis is characterized by altered keratinocyte proliferation and differentiation and by infiltration of activated Th1 and Th17 cells, processes that are linked to altered GATA3 expression, we hypothesized that epithelial GATA3 might play an important role in the pathogenesis of psoriasis. Moreover, psoriasiform epidermal abnormalities, such as epidermal hyperplasia and hyperkeratosis, increased innate immunity and decreased lipid biosynthesis have been reported in mice with an epidermis-specific deletion of GATA3 [9], [10]. We wanted to determine the place of keratinocyte-derived GATA3 during the pathological changes in psoriatic skin. For this we studied different models of psoriasis, such as the imiquimod-induced psoriasis-like skin inflammation in mice [22] and *ex vivo*-stimulated human skin explants, and investigated GATA3 expression and activation in keratinocytes during epidermal regeneration, which is known to share many characteristics with psoriatic skin inflammation [23]. Furthermore, we compared gene expression changes in psoriasis and in epidermis-specific GATA3-knock-out mice, and searched for factors that can correct the downregulated GATA3 expression in psoriasis.

## Materials and Methods

### Ethics statement

Written approval was obtained for all human and animal experimental work. The work protocol including patients with psoriasis was approved by the Medical Ethical Committee of the Erasmus University Medical Center Rotterdam, the Netherlands, approval number was METC 234.237/2003/210. Collection of skin samples after breast reduction surgery was approved by the Medical Ethical Committee of the Erasmus University Medical Center Rotterdam, the Netherlands, approval number was METC 140.050/SPO/1990/30 and 140.050, MEC99.785. Written informed consent was obtained from all patients and healthy subjects involved in the study. All animal work was approved by the Animal Ethical Committee of the Erasmus University Medical Center Rotterdam, the Netherlands, under approval number DEC EUR 851 (OZP 128-06-07). All these approvals were written.

### Patients, NB-UVB treatment and biopsy samples

Sixteen patients (10 men, 6 women, age range 20–73) with psoriasis were recruited after written informed consent (METC registration number 234.237/2003/210). All patients had a Psoriasis Area and Severity Index (PASI) scores of at least 10, no systemic therapy for at least one month or topical therapy for at least two weeks prior to the start of the study. Ten patients were treated with standard NB-UVB phototherapy until total clearance of psoriasis was reached, or for a maximum of three months. From 10 of these patients three-mm biopsies were taken from lesional and non-lesional skin before the start of NB-UVB therapy and after the last treatment session. When PASI scores reached a reduction of 50% of the baseline score, additional biopsies from lesional and non-lesional skin were taken. NB-UVB treatment was applied three times weekly using a Waldmann 7001 UVB cabinet equipped with Philips TL-01 bulbs. Starting UVB dose was 0.1–0.3 J/cm^2^ (depending on the skin type of the patient); the mean cumulative UVB dose was 42.0 J/cm^2^ (range 30–60 J/cm^2^). During the course of UVB treatment PASI scores were evaluated every two weeks.

From six patients biopsy samples were taken from non-lesional skin, from which the stratum corneum was removed by tape stripping [24], [25], [26]. Five hours after tape stripping biopsies were taken from the tape-stripped area.

### Skin organ culture

Skin biopsies (3 mm diameter) were obtained, after informed consent, from healthy volunteers undergoing breast reduction in the Department of Plastic Surgery of the Sint Franciscus Gasthuis, Rotterdam, the Netherlands. Biopsies were cultured in a transwell system as described previously, with the dermis immersed in medium, and the epidermis exposed to the air interface [27]. Recombinant human IL-4 (100 ng/ml, Peprotech, Rocky Hill, New Jersey), IFN-α (500 U/ml), IFN-γ (500 U/ml) or IL-22 (50 ng/ml) (all R&D Systems, Abingdon, UK) were added to the culture medium. These concentrations were previously demonstrated to yield optimal biological responses in this organ culture system. Biopsies were collected 24 h later.

### Keratinocyte culture

Primary human epidermal keratinocytes were obtained from healthy donors as described previously [28] and cultured in Dermalife medium (LifeLine Cell Technology, Walkersville, MD) and transferred to Lab-Tek Chamber slides. Keratinocytes from passages 3 to 4 were used. Cells were cultured with or without CaCl_2_ (final Ca^2+^ concentration was 1.2 mM). High Ca^2+^ conditions were applied in order to stimulate differentiation. In addition, TGF-β1 was added to the cells at a concentration of 3 ng/ml. Keratinocytes were harvested after 24 h, fixed and stained with anti-GATA3 antibody (1∶100; Santa Cruz Biotechnology, Santa Cruz, CA).

### RNA isolation

The epidermis was separated from the dermis after incubation in 1 mg/ml protease X (Sigma Aldrich, Zwijndrecht, the Netherlands) for 90 min at 37°C, and stored in RNA lysis buffer at −80°C until further processing. Total messenger RNA was isolated from the epidermis only, using GenElute Mammalian Total RNA Miniprep kit (Sigma Aldrich). RNA purity and integrity was verified by scanning with an Agilent 2100 Bioanalyzer using RNA 6000 Nano LabChip.

### Array hybridization and analysis

For hybridization on gene expression arrays, RNA samples of individual patients were pooled. Patients were divided into two groups in order to have duplicate arrays for each time point and condition. Biotinylated target RNA was prepared from the pooled (1 µg) total RNA, and hybridized on GeneChip Human Genome U133 Plus 2.0 arrays (Affymetrix, Santa Clara, CA). Array hybridization and scanning was performed as described previously [29]. The data were read and robust multichip analysis (RMA) [30] was used to remove the background and normalize the data across arrays [31]. These values were log_2_-transformed for further analysis, yielding numbers between 0 and 16. A two-way ANOVA with factors “probe” and “condition” was used for each probeset to calculate both average expression levels per condition [32] and a *p*-value for the difference between conditions. The resulting *p*-values were adjusted for multiple testing using Šidák step-up adjustment [33]. Genes were considered differentially expressed when *p*-values were <0.05.

Gene expression analysis of isolated hair follicles of GATA3−/− mice was perfomed as described previously [9]. Microarray data complied with the MIAME regulations and are available in ArrayExpress (accession code: 5988). Functional annotation was performed using the Database for Annotation, Visualization, and Integrated Discovery (DAVID) [34].

### Quantitative RT-PCR

RNA of individual patients was transcribed into cDNA, and RT-PCR was performed as described previously [35]. ABL1 was used as a housekeeping control gene. Sequences of newly designed primers and probe numbers of the Exiqon probe library system (Exiqon, Vedbaek, Denmark) are listed in Table 1.

**Table 1:** Primers and probes for RT-PCR.

Gene	Forward primer	Reverse primer	Probe
**hGATA3**	GCTTCGGATGCAAGTCCA	GCCCCACAGTTCACACACT	Nr. 8^1^
**mGATA3**	CATTACCACCTATCCGCCCTATG	CACACACTCCCTGCCTTCTGT	CGAGGCCCAAGGCACGATCCAG
**hGAPDH**	TCCACTGGCGTCTTCAC	GGCAGAGATGATGACCCTTTT	Nr. 45^1^
**h/mABL1**	TGGAGATAACACTCTAAGCATAACTAAAGGT	GATGTAGTTGCTTGGGACCCA	CCATTTTTGGTTTGGGCTTCACACCATT

1 Probe from the Exiqon probe library system (Exiqon, Vedbaek, Denmark).

### Mice and treatments

Induction of psoriasiform skin inflammation by application of imiquimod on BALB/c and LacZ knock in GATA3 mice [36] was performed as described previously [22]. Briefly, mice were treated daily with imiquimod on the shaved back skin for 5 days. Every other day, starting on the first day of the experiment, mice were irradiated with a Waldmann NB-UVB irradiation device equipped with TL-01 UV 236-01 lamps (Waldmann Medizintechnik, Villingen-Schwenningen, Germany), or were sham irradiated. The applied starting UVB dose was 70% of the minimal erythema dose (MED) and it was increased by 10% each treatment. Scoring of the severity of skin inflammation was performed as described previously [22]. On day 6, mice were sacrificed and 3 mm biopsy samples were taken from the back skin. Total RNA was isolated, transcribed into cDNA, and the expression of GATA3 was determined by RT-PCR, using ABL1 as a housekeeping control gene.

To determine the MED in BALB/c mice, animals were irradiated with increasing doses of NB-UVB. Ear thickness was measured with a micrometer (Mitutoyo, Veenendaal, the Netherlands) before the UVB irradiation and 48 h later. The lowest NB-UVB dose where ear thickness was significantly increased was 1680 mJ/cm^2^. 70% of this dose (thus 70% of the MED) was used as the starting dose in further experiments.

Prior to the irradiation experiment one of the LacZ knockin GATA3 mice was critically bitten and had to be sacrificed. We took advantage of this unintentional skin wounding to assess whether this insult and concomitant skin regeneration affected GATA3 expression. The wounded back skin of this mouse as well as unaffected adjacent skin was embedded in Tissue-Tec embedding medium (Sakura, Zoeterwoude, the Netherlands) and snap-frozen.

### Immunohistochemistry, X-Gal staining and BrdU labelling

For immunofluorescent staining, cryosections or cells were fixed for 10 min in 4% paraformaldehyde (PFA) in PBS. Primary antibodies included anti-β-defensin 2 (1∶100; Santa Cruz Biotechnology, Santa Cruz, CA), anti-GATA3 (1∶100; Santa Cruz Biotechnology), anti-phosphorylated STAT3 (1∶50; Cell Signaling Technology Inc, Danvers, MA) and K10 (mouse 1∶50; Sigma, clone k8.60). Relevant FITC-, TxR- or HRP-conjugated antibodies (1∶100, Abcam) were used to detect primary antibodies. All fluorescent images were taken with an Axio Imager (Zeiss) fluorescence microscope.

Mice were injected with 50 mg/kg bodyweight 5-bromo-2′-deoxyuridine (BrdU) and sacrificed 2 h later. Cryosections were fixed for 10 min in 4% PFA in PBS and washed three times for 5 min in PBS. As block/diluent was used: 1% BSA, 0.05% Tween in PBS. For BrdU immunohistochemistry with BrdU (mouse 1∶100; DAKO, clone Bu20a), tissue samples were fixed in 4% PFA in PBS at 4°C overnight. Skin samples were subsequently embedded in paraffin and sectioned at 5 µm. After deparaffination sections were boiled in 0.01 M citrate buffer (pH 6.0) for 15 min prior to incubation with primary antibody. Sections were analyzed and photographed with an Olympus BX40 light microscope.

For X-gal staining sections were fixed 1 min in 0.5% glutaraldehyde, 1% PFA, washed in PBS and incubated in X-gal staining solution (5 mM K_3_Fe(CN)_6_, 5 mM K_4_Fe(CN)_6*_3H_2_0, 2 mM MgCl_2_, 0.01% natrium-deoxycholate, 0.02% NP40, 1 mg/ml bromo-chloro-indolyl-galactopyranoside) for 5 h at room temperature. Sections then were fixed in 4% PFA for 10 min and counterstained with neutral red.

## Results

### Epidermal GATA3 expression is decreased in human psoriatic lesions and in psoriasiform dermatitis in mice

**Figure 1:**
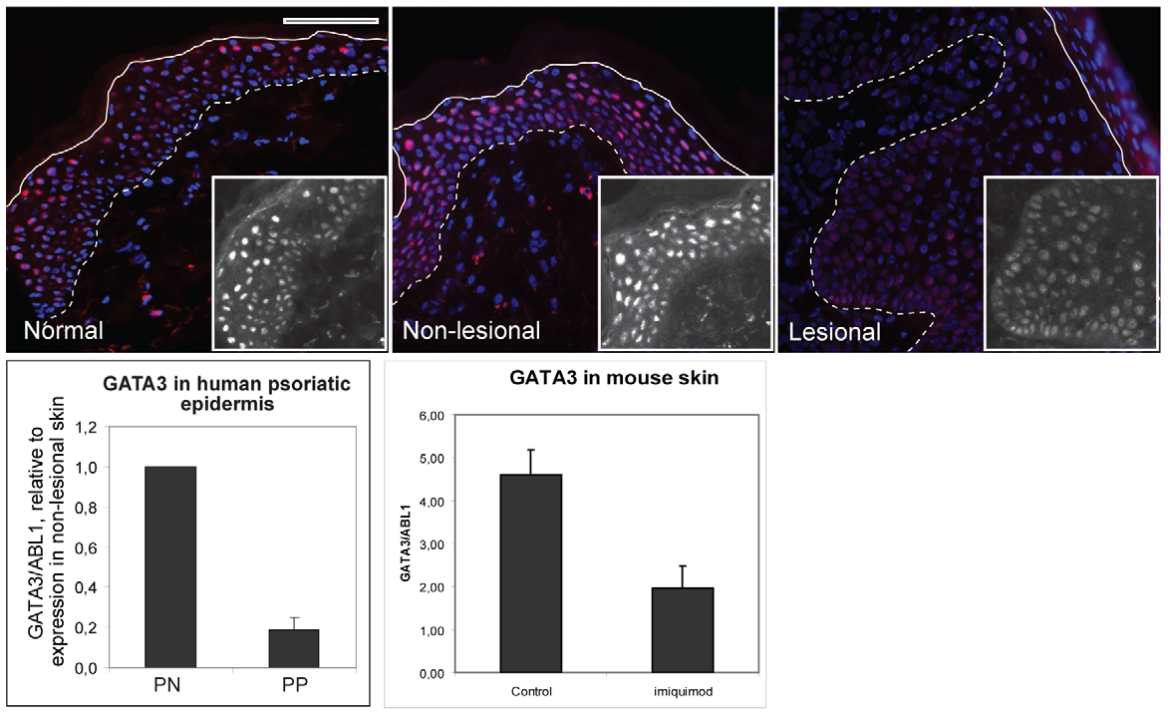
Epidermal GATA3 expression is decreased in psoriatic lesions. **A.** GATA3 protein is present in the nuclei of the suprabasal layers of the epidermis in normal skin. In non-lesional psoriatic skin GATA3 is expressed in all epidermal layers whereas in lesional skin sporadic GATA3 expression is observed. The borders of the epidermis are depicted. Scale bar: 100 µm. **B.** Expression of GATA3 mRNA was almost seven-fold lower in lesional skin (PP) compared to non-lesional skin (PN). Epidermal GATA3 mRNA expression was determined by RT-PCR using ABL1 as a housekeeping control gene. In each patient, GATA3 expression in lesional skin is calculated relative to the expression in non-lesional skin. Bars represent mean +/− SEM (n = 5 patients). **C.** GATA3 mRNA expression in imiquimod-induced psoriasiform dermatitis in mice is lower than in control back skin of BALB/c mice. Bars indicate the mean +/− SEM (n = 3 mice per group).

Previous studies showed that GATA3 is downregulated in lesional psoriatic epidermis ([4], Rácz et al, submitted). To validate this finding, GATA3 mRNA expression was measured by quantitative RT-PCR in the epidermis of skin biopsies from patients with psoriasis and skin from healthy controls. A 5-fold (range 3–12-fold) lower expression of GATA3 was observed in lesional epidermis compared to non-lesional epidermis (Figure 1B). GATA3 expression was also determined *in situ* at the protein level, in skin biopsies from healthy individuals, as well as in lesional and non-lesional skin biopsies of patients with psoriasis. In healthy control skin GATA3 expression was present in all epidermal layers except the basal layer (Figure 1A). In non-lesional psoriatic skin, GATA3 expression was also visible in the basal layer, while in lesional psoriatic skin overall GATA3 expression was decreased and GATA3 was only present at low level in a few suprabasal cells (Figure 1A).

Next we assessed the expression of GATA3 in imiquimod-induced psoriasiform dermatitis in mice. Daily application of the TLR7/8 agonist imiquimod cream (Aldara®) on mouse back skin induces skin inflammation that strongly resembles human psoriasis in terms of phenotypic and histological features [22]. Mean relative GATA3 mRNA expression as measured by RT-PCR, using ABL1 as a control housekeeping gene, was 4.60 in the control mice (range 3.10–9.22, n = 5), whereas in mice treated with imiquimod mean relative GATA3 expression was 1.96 (range 1.16–3.26, n = 5). Thus, GATA3 mRNA expression measured by RT-PCR was 2.35-fold lower in the psoriasiform dermatitis lesions than in control mouse skin (*p* = 0.016, determined using the Mann-Whitney U test) (Figure 1C).

### GATA3 expression in psoriasis is upregulated by effective treatment of the disease

**Figure 2:**
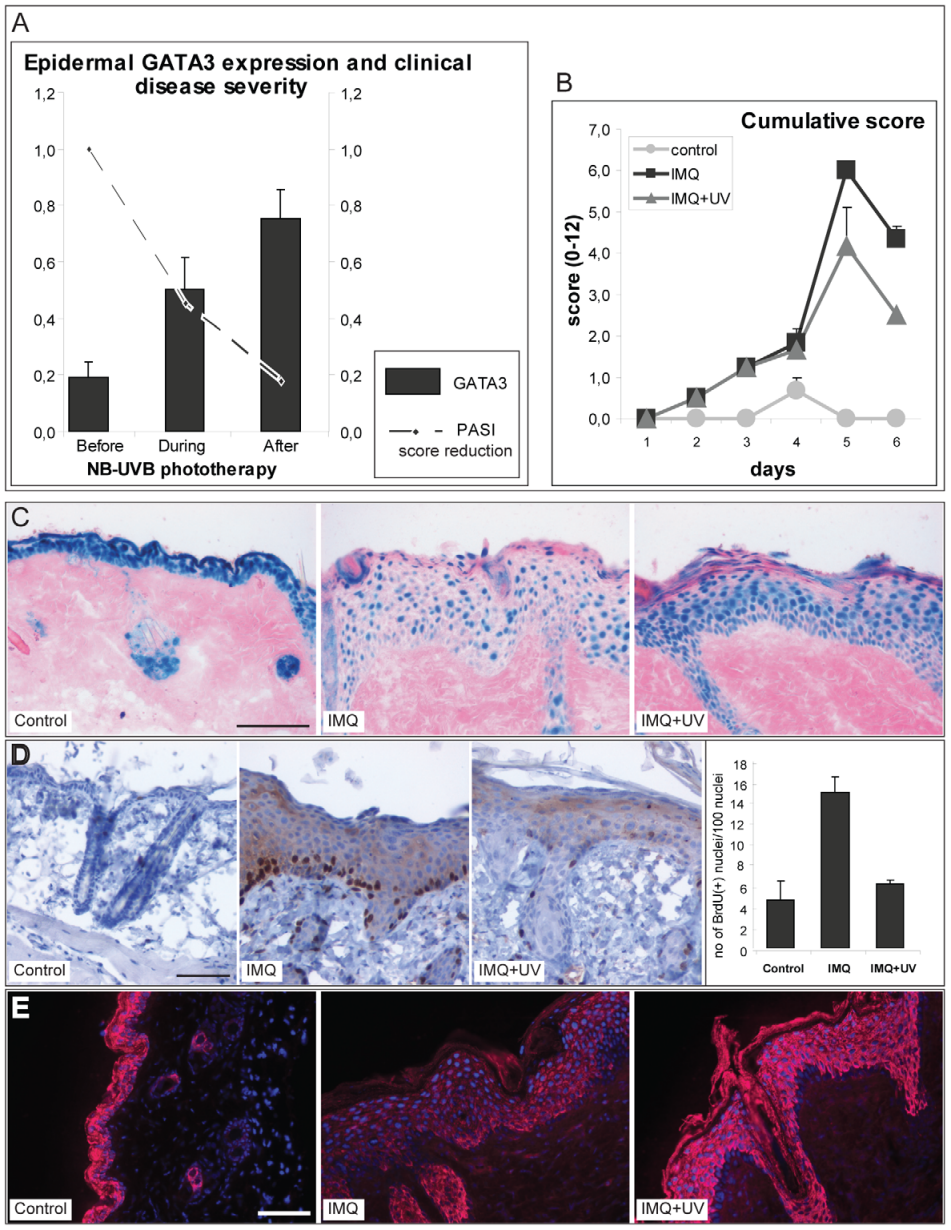
NB-UVB phototherapy induces GATA3 expression. **A.** GATA3 expression is upregulated during the course of NB-UVB phototherapy in patients with psoriasis. 3 mm biopsies were taken from lesional (PP) and non-lesional skin of patients with psoriasis before, during and after NB-UVB phototherapy. Epidermal GATA3 mRNA expression was determined with RT-PCR using ABL1 as a housekeeping control gene. Bars represent mean +/− SEM (n = 5 patients). The interrupted line shows change in the PASI score, relative to the baseline score. **B.** Psoriasiform dermatitis was induced in BALB/c mice by daily treatment with IMQ cream (or control cream) on the shaved back skin, and irradiated or sham-irradiated every other day with NB-UVB, starting on the first day of imiquimod treatment. Erythema, scaling, and thickness of the back skin were scored daily on a scale from 0 to 4. The cumulative score (erythema plus scaling plus thickness) is shown. Symbols indicate mean score +/− SEM of three mice per group. **C.** X-gal staining (blue) of GATA3LacZ skin biopsies from mice with psoriasiform dermatitis with or without NB-UVB treatment. **D.** BrdU incorporation in keratinocytes in the back skin was detected by immunohistochemistry. The bars represent the mean number of BrdU positive cells +/− SD. **E.** Biopsies from psoriasiform dermatitis on the back skin of the mice were stained for Keratin 1/10. Scale bars on panels C, D and E: 100 µm.

NB-UVB phototherapy is a standard and effective treatment modality for human psoriasis. We evaluated whether NB-UVB treatment of patients with psoriasis would induce epidermal GATA3 expression towards the levels in normal skin. We therefore measured GATA3 expression by RT-PCR in patients with psoriasis undergoing standard NB-UVB therapy. Indeed, GATA3 mRNA expression increased gradually during the course of NB-UVB treatment, correlating inversely with the clinical PASI score (Figure 2A).

Similarly, NB-UVB treatment also inhibited the severity of the psoriasiform dermatitis in mice by approximately 40% (*p* = 0.034 as determined with the Mann-Whitney U test, n = 3 mice per group) (Figure 2B). Accordingly, histological examination showed decreased epidermal thickness, improved epidermal differentiation and a significantly reduced number of proliferating BrdU^+^ cells in NB-UVB-treated as compared to control mouse skin (Figure 2D, E). NB-UVB treatment clearly reduced markers of psoriasis activity such as epidermal phosphorylated STAT3, dendritic cell and neutrophilic granulocyte infiltrates and angiogenesis [5]. Thus, NB-UVB irradiation of imiquimod-treated mouse skin results in improvement of psoriasiform dermatitis, as assayed by clinical, histological and immunohistochemical parameters.

We next assessed GATA3 expression during NB-UVB irradiation of murine psoriasiform dermatitis. Therefore psoriasiform dermatitis was induced in GATA3-LacZ knockin mice [36], followed by irradiation every other day with NB-UVB or sham UVB treatment. In these mice, the expression of the LacZ product X-Gal depends on GATA3 promoter activity. On day 6 mice were sacrificed and GATA3 expression was assayed by X-Gal staining of back skin sections. X-Gal staining was decreased in the psoriasiform mouse skin, and was clearly re-induced by NB-UVB irradiation (Figure 2C). In conclusion, successful treatment of psoriasis patients and of murine psoriasiform skin inflammation is associated with upregulation of GATA3 expression, which parallels clinical improvement.

### GATA3 expression is downmodulated during epidermal regeneration

**Figure 3:**
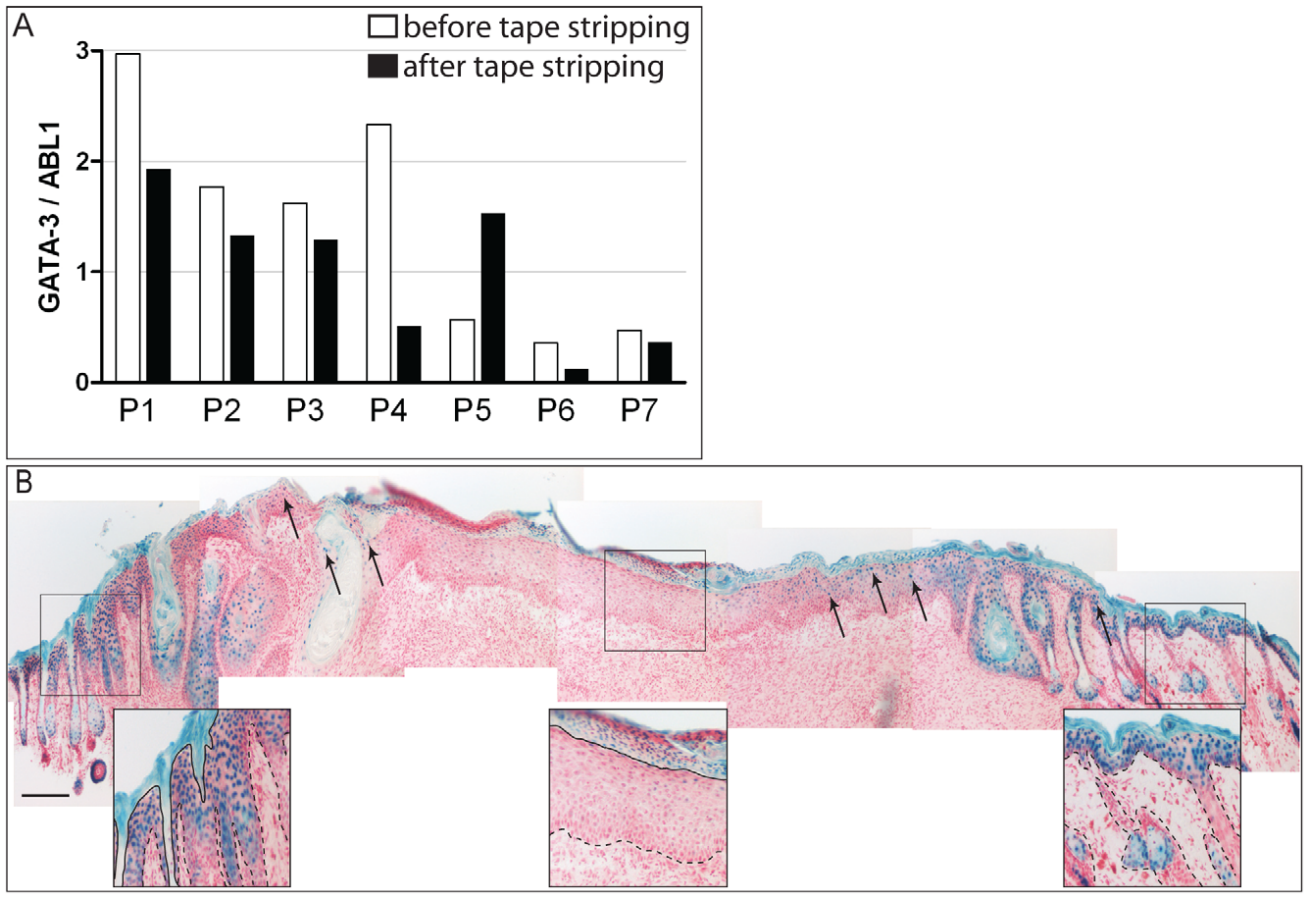
GATA3 expression is decreased in the regenerating epidermis. **A.** Six psoriatic patients underwent repeated tape stripping to induce skin activation and regeneration. Directly before and 5 h after tape stripping biopsies were taken and epidermal GATA3 expression was determined using RT-PCR, relative to ABL1 as a housekeeping control gene. Relative expression values of individual patients are shown. **B.** GATA3 expression is downregulated in the healing wound of mouse skin. X-gal staining (blue) of wounded skin of a GATA3LacZ mouse shows downregulation of the LacZ transgene under control of GATA3 in the highly proliferative zone of the healing wound. Arrows indicate X-Gal positive cells; the border between regenerating and adjacent normal skin is marked. Scale bar: 200 µm.

The epidermal response in psoriasis shares many similarities with the epidermal regeneration program upon wounding [23]. During epidermal regeneration not only keratinocyte proliferation and dermal angiogenesis are stimulated, but also an inflammatory and antimicrobial response is initiated to prevent infection through the injured epidermal barrier. We asked whether decreased keratinocyte GATA3 expression was specific to psoriatic inflammation or could also be seen during epidermal regeneration. First, epidermal barrier disruption was induced in non-lesional skin of patients with psoriasis by repeated tape stripping. Before tape stripping and 5 h later, skin biopsies were taken from the tape-stripped area, the epidermis was separated from the dermis, and mRNA expression of epidermal GATA3 quantitated. Skin tape stripping downregulated GATA3 expression in the epidermis by a mean of 80% from baseline levels (*p* = 0.03 using the Wilcoxon signed rank test, n = 6 patients) (Figure 3A).

We additionally investigated GATA3 expression during wound healing by performing X-Gal staining on sections taken from the wounded back skin of GATA3 LacZ knock in mice. GATA3 was expressed in all epidermal layers of the intact epidermis, whereas in the center of the wound only few X-Gal positive keratinocytes were seen (Figure 3B). In conclusion, GATA3 expression is downmodulated in the regenerating epidermis after wounding and tape stripping, and in psoriasis.

### GATA3 downregulation is associated with decreased expression of TNFAIP3, Jagged and AP2 transcription factors in keratinocytes

To identify other transcription factors associated with downmodulated GATA3 expression in psoriatic epidermis, we compared the gene expression profiles from epidermis-specific GATA3-deficient mice [9] with that of lesional epidermis of patients with psoriasis. For this, two different comparisons were made: A: RNA from mouse hair follicles of wild type versus GATA3−/− mice. B: RNA from human epidermis from non-lesional versus lesional skin. After this, the two lists of differentially expressed genes were crossed, thereby selecting genes that were either down- or up-regulated both in GATA3−/− epidermis (when compared to WT mouse epidermis), as wells as in lesional psoriatic epidermis (when compared to non-lesional epidermis).

In the epidermis of epidermis-specific GATA3−/− mice 5922 genes were differentially expressed when compared to wild type mice (p<0.05, ≥1.2-fold up- or downregulated [9]). In human lesional psoriatic epidermis, characterized by low GATA3 expression, 2412 genes were differentially expressed when compared to non-lesional epidermis (p<0.05, ≥1.2-fold up- or downregulated) [5]. Genes were identified that were upregulated in both GATA3-deficient mouse skin and in lesional psoriatic skin (77 genes), or were downregulated in both (97 genes) (Table 2, Table S1). The resulting group of genes included negative regulators of inflammation, such as the psoriasis susceptibility gene TNFAIP3 [21], [37] and the Notch ligand jagged 2 [38], and also genes regulating epidermal differentiation, such as the transcription factor AP2-α (TFAP2A) [39], and the apoptosis-inducing FAS molecule. Expression of several other transcription factors was regulated in a coordinated fashion with GATA3 (Table S1), e.g. FOXP1, FOXO1, FOXN3, KLF13 and SNAI2, factors mainly involved in organ development and differentiation.

**Table 2:** List^1^ of genes differentially expressed in both psoriasis and GATA3 −/− mice.

Symbol	Cell differentiation	Fold change human	Fold change mouse	Up/down
SOD2	SUPEROXIDE DISMUTASE 2, MITOCHONDRIAL	6.0	1.2	U
ALDH1A3	ALDEHYDE DEHYDROGENASE 1 FAMILY, MEMBER A3	3.9	1.5	U
TXNDC5	THIOREDOXIN DOMAIN CONTAINING 5	2.5	1.3	U
EHF	ETS HOMOLOGOUS FACTOR	2.3	1.3	U
CTSB	CATHEPSIN B	2.1	1.3	U
	**Apoptosis**			
SOD2	SUPEROXIDE DISMUTASE 2, MITOCHONDRIAL	6.0	1.2	U
ALDH1A3	ALDEHYDE DEHYDROGENASE 1 FAMILY, MEMBER A3	3.9	1.5	U
TXNDC5	THIOREDOXIN DOMAIN CONTAINING 5	2.5	1.3	U
CTSB	CATHEPSIN B	2.1	1.3	U
TNFAIP3	TUMOR NECROSIS FACTOR, ALPHA-INDUCED PROTEIN 3	2.6	1.3	D
	**Transcription regulation**			
TSC22D1	TSC22 DOMAIN FAMILY, MEMBER 1	3.0	1.7	D
EHF	ETS HOMOLOGOUS FACTOR	2.3	1.3	U
HLF	HEPATIC LEUKEMIA FACTOR	2.3	1.5	D
NFIB	NUCLEAR FACTOR I/B	2.3	1.4	D
KAT2B	K(LYSINE) ACETYLTRANSFERASE 2B	2.2	1.7	D
	**Other**			
DSC2	DESMOCOLLIN 2	4.0	1.9	U
TF	TRANSFERRIN	2.2	2.0	U
LNX1	LIGAND OF NUMB-PROTEIN X 1	4.3	1.6	D
INSIG2	INSULIN INDUCED GENE 2	2.9	1.4	D
IGFBP5	INSULIN-LIKE GROWTH FACTOR BINDING PROTEIN 5	2.7	1.3	D

1 Top five genes per functional category are listed. For the complete list see Table S1.

Additionally, the list of 174 genes was subjected to gene annotation using the DAVID Gene Annotation Tool. This comparison showed that low GATA3 expression in both human psoriatic and GATA3-deficient murine epidermis coincided with altered expression of genes involved in cell differentiation, cell proliferation and apoptosis (Table 2, Table S1).

### GATA3 in proliferating, differentiating and cell cycle arrested keratinocytes

**Figure 4:**
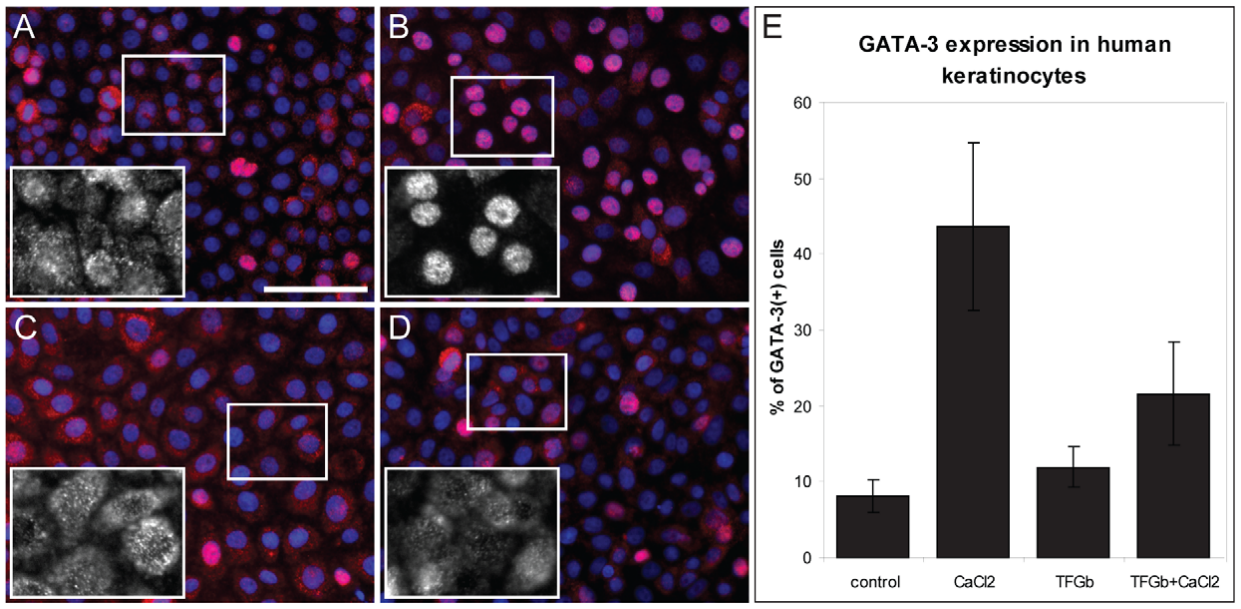
Nuclear translocation of GATA3 in differentiating keratinocytes, but not during cell cycle arrest. Primary human epidermal keratinocytes were cultured on chamber slides. When cells were approximately 75% confluent, 1.2 mM CaCl_2_ (**B**, **D**) and/or 3 ng/ml TGF-β1 (**C**, **D**) were added to the culture medium. After 24 h cells were fixed and immunofluorescent staining for GATA3 protein (pink) was performed. **E.** GATA3 positive cell nuclei were counted and are shown as a percentage of the total number of cell nuclei. Scale bar: 20 µm.

The expression of GATA3 was investigated in relationship to keratinocyte differentiation and proliferation by immunofluorescent staining of undifferentiated, growth-arrested and calcium-induced differentiated keratinocytes. In differentiating keratinocytes GATA3 staining was localized only in the nucleus, whereas in normal proliferating keratinocytes GATA3 staining was present in the nuclei and in the cytoplasm (Figure 4A, B, D). In addition, cell cycle arrest was induced in keratinocytes by culturing them in the presence of 3 ng/ml TGF-β1 at low and high Ca2+ conditions. Interestingly, GATA3 staining in TGF-β1-treated cells was almost completely localized to the cytoplasm, indicating lack of signaling (Figure 4C,D).

In conclusion, in differentiated keratinocytes GATA3 is transcriptionally active, whereas no nuclear GATA3 could be observed during cell cycle arrest, indicating that GATA3 is important during keratinocyte differentiation and proliferation.

### The Th2 cytokine IL-4 induces GATA3 in human epidermal cells

**Figure 5:**
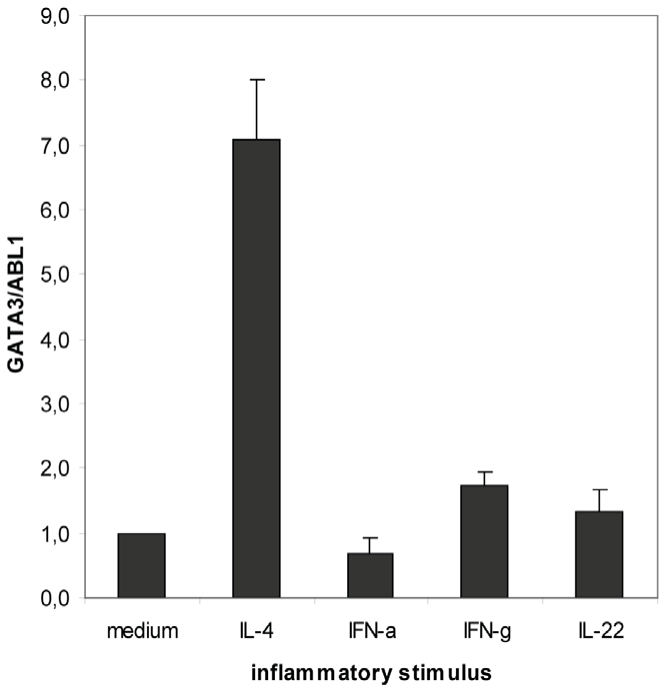
IL-4 induces the expression of GATA3 in human epidermis. Three mm biopsies from normal human skin were cultured in the presence of proinflammatory cytokines for 24 h. The epidermis was separated from the dermis and GATA3 expression was measured by RT-PCR in epidermal RNA, using ABL1 as a housekeeping control gene. Bars represent mean +/− SEM (n = 4 (IFN-α, IL-22), n = 6 (IL-4) or n = 8 (IFN-γ) healthy donors).

We next asked which cytokines in psoriatic lesions could induce or downmodulate GATA3. We therefore stimulated skin biopsies from healthy volunteers ex vivo with different cytokines important in the pathogenesis of psoriasis and measured epidermal GATA3 mRNA. Human skin biopsies were cultured with IFN-α, [40], IFN-γ, or IL-22, prototypic Th1 and Th17 cytokines, respectively, and the prototypic Th2 cytokine IL-4. IL-4 caused a 7-fold induction of GATA3 expression (range 4.2–10.7, n = 6 healthy donors). IFN-γ slightly induced GATA3 expression (1.7-fold; range 1.2–2.7, n = 8 healthy donors), whereas IFN-α and IL-22 had no significant effect (Figure 5). TNF-α, IL-17A, IL-23, IL-1β, oncostatin M, nerve growth factor (NGF), substance P and combinations of these also did not induce or suppress GATA3 (*data submitted*).

## Discussion

Our results show that the epidermal expression of the transcription factor GATA3 is consistently decreased in psoriasis, in psoriasiform dermatitis in mice, and during epidermal wound healing. Our finding that epidermal GATA3 expression is downmodulated in both psoriasis and epidermal regeneration is consistent with previously described parallels between these conditions. It identifies GATA3 as a crucial transcription factor in keratinocyte homeostasis, activation and proliferation. We also show that in healthy human skin explants the Th2 cytokine IL-4 was the only cytokine, out of a broad array of cytokines critical in psoriasis, able to significantly and effectively induce GATA3 expression in the epidermis.

Although downmodulated GATA3 levels have been reported in microarray studies of psoriasis [4], [41], [42], its role and modulation of its expression in human skin was not investigated in great detail. Polymorphisms in the GATA3 gene or genetic linkage have not been reported for psoriasis, but have been found for atopic dermatitis. The latter association is not unexpected since this chronic inflammatory skin disease is linked to a Th2 signature [43].

Most of our current insight into the function of GATA3 originates from T cell biology, whereas much less is known about GATA3 molecular function in keratinocytes. In T cells GATA3 determines Th2 cell differentiation and selectively activates the promoters of IL-4, IL-5, and IL-13 through chromatin remodelling. For the latter, GATA3 must translocate from the cytoplasm into the nucleus to access its target genes. Here we show that in keratinocytes GATA3 is strongly induced by IL-4. Psoriasis is currently viewed as a predominantly Th1/Th17 disease, where the Th1 cytokines (IFN-γ) and Th17 cytokines (IL-17, IL-22) together with IL-23 suppress the production of IL-4 in T lymphocytes. Perhaps the relative lack of IL-4 in psoriatic lesions contributes to the reduction of GATA3 expression in the epidermis (and also to the upregulation of the IL-4 receptor on psoriatic keratinocytes [44]). In a clinical trial IL-4 was previously shown to effectively clear psoriasis [45]. In spite of the promising results, IL-4 was, not further developed into clinical practice for commercial reasons.

At the time, the beneficial effect of IL-4 in psoriasis was attributed to the Th2-inducing and Th1 inhibitory capacity of IL-4 together with modulation of IL-23 production by dermal inflammatory APC [46]. Our results provide a novel mechanism in keratinocytes by which IL-4 improves psoriasis.

To identify molecules that are co-ordinately regulated with GATA3 in psoriasis, we determined the overlap between genes differentially regulated in epidermis-specific GATA3 deficient mice and genes that were differentially expressed in human lesional psoriatic skin compared to non-lesional skin. The resulting group of genes included negative regulators of inflammation, such as the psoriasis susceptibility gene TNFAIP3 [21], [37] and the Notch ligand jagged 2 [38], and also genes regulating epidermal differentiation, such as the transcription factor AP2-α (TFAP2A) [39], and the apoptosis-inducing FAS molecule. In addition, the transcription factors FOXN3 and FOXO1, regulating cell differentiation and the cell cycle, were also downregulated together with GATA3. These molecules play regulatory roles in organ development [47], metabolism [48] and cell proliferation [49], respectively. Together with the induction of GATA3, AP2-α (TFAP2A), the anti-inflammatory TNFAIP3 and Jagged 2 molecules were also induced in lesional psoriatic skin by NB-UVB. Induction of these molecules might contribute to the anti-psoriatic effects of NB-UVB.

In conclusion, this study shows that the epidermal expression of the transcription factor GATA3 is consistently downregulated under conditions of keratinocyte hyperproliferation and altered differentiation such as in psoriasis, in murine psoriasiform dermatitis and in wound healing. Our results indicate that induction of GATA3 expression in keratinocytes may be a novel therapeutic strategy in the treatment of psoriasis.

## Supporting Information

Table S1 **List of genes differentially expressed in both psoriasis and GATA3 −/− mice.**
(DOC)
