# Supplementary material for: GATA3 Expression Is Decreased in Psoriasis and during Epidermal Regeneration; Induction by Narrow-Band UVB and IL-4
Source: PLoS One. 2011 May 17;6(5):e19806. doi: 10.1371/journal.pone.0019806 (PMC3096641; doi:10.1371/journal.pone.0019806)
Supplement: Table S1 — List of genes differentially expressed in both psoriasis and GATA3 −/− mice. (DOC) [file pone.0019806.s001.doc]

**Table S1. List of genes differentially expressed in both psoriasis and GATA3 -/- mice.**

| **Symbol** | **Cell differentiation** | **Fold change human** | **Fold change mouse** | **Up/down** |
| --- | --- | --- | --- | --- |
| SOD2 | SUPEROXIDE DISMUTASE 2, MITOCHONDRIAL | 6.0 | 1.2 | U |
| ALDH1A3 | ALDEHYDE DEHYDROGENASE 1 FAMILY, MEMBER A3 | 3.9 | 1.5 | U |
| TXNDC5 | THIOREDOXIN DOMAIN CONTAINING 5 | 2.5 | 1.3 | U |
| EHF | ETS HOMOLOGOUS FACTOR | 2.3 | 1.3 | U |
| CTSB | CATHEPSIN B | 2.1 | 1.3 | U |
| S100A6 | S100 CALCIUM BINDING PROTEIN A6 (CALCYCLIN) | 1.8 | 1.4 | U |
| SGK1 | SERUM/GLUCOCORTICOID REGULATED KINASE | 1.8 | 2.4 | U |
| EIF2B2 | EUKARYOTIC TRANSLATION INITIATION FACTOR 2B, SUBUNIT 2 BETA | 1.7 | 1.7 | U |
| DICER1 | DICER1, RIBONUCLEASE TYPE III | 1.6 | 1.6 | U |
| POLB | POLYMERASE (DNA DIRECTED), BETA | 1.6 | 1.4 | U |
| PHB | PROHIBITIN | 1.6 | 1.4 | U |
| BOK | BCL2-RELATED OVARIAN KILLER | 1.4 | 1.5 | U |
| TXNL1 | THIOREDOXIN-LIKE 1 | 1.3 | 1.3 | U |
| CHL1 | CELL ADHESION MOLECULE WITH HOMOLOGY TO L1CAM | 4.5 | 2.0 | D |
| TNFAIP3 | TUMOR NECROSIS FACTOR, ALPHA-INDUCED PROTEIN 3 | 2.6 | 1.3 | D |
| TNFRSF19 | TUMOR NECROSIS FACTOR RECEPTOR SUPERFAMILY, MEMBER 19 | 2.3 | 1.4 | D |
| FAS | FAS (TNF RECEPTOR SUPERFAMILY, MEMBER 6) | 2.0 | 1.5 | D |
| DLG5 | DISCS, LARGE HOMOLOG 5 | 1.9 | 1.6 | D |
| FOXO1 | FORKHEAD BOX O1A | 1.9 | 1.6 | D |
| MYST3 | MYST HISTONE ACETYLTRANSFERASE 3 | 1.9 | 1.5 | D |
| TNFRSF25 | TUMOR NECROSIS FACTOR RECEPTOR SUPERFAMILY, MEMBER 25 | 1.9 | 1.2 | D |
| RYBP | RING1 AND YY1 BINDING PROTEIN | 1.8 | 1.5 | D |
| SPRY1 | SPROUTY HOMOLOG 1, ANTAGONIST OF FGF SIGNALING | 1.8 | 1.5 | D |
| MIB1 | MINDBOMB HOMOLOG 1 | 1.7 | 1.4 | D |
| PDCD6IP | PROGRAMMED CELL DEATH 6 INTERACTING PROTEIN | 1.6 | 1.4 | D |
| UBE2B | UBIQUITIN-CONJUGATING ENZYME E2B | 1.6 | 1.5 | D |
| JAG2 | JAGGED 2 | 1.5 | 1.5 | D |
| DHCR24 | 24-DEHYDROCHOLESTEROL REDUCTASE | 1.2 | 2.1 | D |
|  |  |  |  |  |
|  | **Cell cycle** |  |  |  |
| KLK10 | KALLIKREIN 10 | 2.3 | 2.0 | U |
| S100A6 | S100 CALCIUM BINDING PROTEIN A6 (CALCYCLIN) | 1.8 | 1.4 | U |
| GSPT1 | G1 TO S PHASE TRANSITION 1 | 1.7 | 1.4 | U |
| PHB | PROHIBITIN | 1.6 | 1.4 | U |
| AURKAIP1 | AURORA KINASE A INTERACTING PROTEIN 1 | 1.5 | 1.4 | U |
| PA2G4 | PROLIFERATION-ASSOCIATED 2G4 | 1.5 | 1.3 | U |
| MCC | MUTATED IN COLORECTAL CANCERS | 3.0 | 1.5 | D |
| WEE1 | WEE1 HOMOLOG | 2.5 | 1.5 | D |
| KAT2B | K(LYSINE) ACETYLTRANSFERASE 2B | 2.2 | 1.7 | D |
| FOXN3 | FORKHEAD BOX N3 | 2.1 | 1.5 | D |
| MAP2 | MICROTUBULE/ASSOCIATED PROTEIN 2 | 2.1 | 1.6 | D |
| DLG5 | DISCS, LARGE HOMOLOG 5 | 1.9 | 1.6 | D |
| BUB3 | BUB3 BUDDING UNINHIBITED BY BENZIMIDAZOLES 3 HOMOLOG | 1.6 | 1.6 | D |
| JAG2 | JAGGED 2 | 1.5 | 1.5 | D |
| WNK1 | WNK LYSINE DEFICIENT PROTEIN KINASE 1 | 1.5 | 2.3 | D |
| DHCR24 | 24-DEHYDROCHOLESTEROL REDUCTASE | 1.2 | 2.2 | D |
| CCNI | CYCLIN I | 1.2 | 1.4 | D |
|  |  |  |  |  |
|  | **Apoptosis** |  |  |  |
| SOD2 | SUPEROXIDE DISMUTASE 2, MITOCHONDRIAL | 6.0 | 1.2 | U |
| ALDH1A3 | ALDEHYDE DEHYDROGENASE 1 FAMILY, MEMBER A3 | 3.9 | 1.5 | U |
| TXNDC5 | THIOREDOXIN DOMAIN CONTAINING 5 | 2.5 | 1.3 | U |
| CTSB | CATHEPSIN B | 2.1 | 1.3 | U |
| SGK1 | SERUM/GLUCOCORTICOID REGULATED KINASE | 1.8 | 2.4 | U |
| POLB | POLYMERASE (DNA DIRECTED), BETA | 1.6 | 1.4 | U |
| PHB | PROHIBITIN | 1.6 | 1.4 | U |
| BOK | BCL2-RELATED OVARIAN KILLER | 1.4 | 1.5 | U |
| TXNL1 | THIOREDOXIN-LIKE 1 | 1.3 | 1.3 | U |
| TNFAIP3 | TUMOR NECROSIS FACTOR, ALPHA-INDUCED PROTEIN 3 | 2.6 | 1.3 | D |
| TNFRSF19 | TUMOR NECROSIS FACTOR RECEPTOR SUPERFAMILY, MEMBER 19 | 2.3 | 1.4 | D |
| FAS | FAS (TNF RECEPTOR SUPERFAMILY, MEMBER 6) | 2.0 | 1.5 | D |
| DLG5 | DISCS, LARGE HOMOLOG 5 (DROSOPHILA) | 1.9 | 1.6 | D |
| FOXO1 | FORKHEAD BOX O1A | 1.9 | 1.6 | D |
| TNFRSF25 | TUMOR NECROSIS FACTOR RECEPTOR SUPERFAMILY, MEMBER 25 | 1.9 | 1.2 | D |
| RYBP | RING1 AND YY1 BINDING PROTEIN | 1.8 | 1.5 | D |
| PDCD6IP | PROGRAMMED CELL DEATH 6 INTERACTING PROTEIN | 1.6 | 1.4 | D |
| DHCR24 | 24-DEHYDROCHOLESTEROL REDUCTASE | 1.2 | 2.2 | D |
|  |  |  |  |  |
|  | **Transcription regulation** |  |  |  |
| EHF | ETS HOMOLOGOUS FACTOR | 2.3 | 1.3 | U |
| SUB1 | SUB1 HOMOLOG | 1.8 | 1.4 | U |
| ASCC3 | ACTIVATING SIGNAL COINTEGRATOR 1 COMPLEX 3 | 1.5 | 1.4 | U |
| PA2G4 | PROLIFERATION-ASSOCIATED 2G4 | 1.5 | 1.3 | U |
| FOXP1 | FORKHEAD BOX P1 | 1.4 | 1.4 | U |
| PHB2 | PROHIBITIN 2 | 1.3 | 1.4 | U |
| TSC22D1 | TSC22 DOMAIN FAMILY, MEMBER 1 | 3.0 | 1.7 | D |
| HLF | HEPATIC LEUKEMIA FACTOR | 2.3 | 1.5 | D |
| NFIB | NUCLEAR FACTOR I/B | 2.3 | 1.4 | D |
| KAT2B | K(LYSINE) ACETYLTRANSFERASE 2B | 2.2 | 1.7 | D |
| FOXN3 | FORKHEAD BOX N3 | 2.1 | 1.5 | D |
| FOXO1 | FORKHEAD BOX O1A | 1.9 | 1.6 | D |
| MYST3 | MYST HISTONE ACETYLTRANSFERASE 3 | 1.9 | 1.5 | D |
| SMARCA2 | SWI/SNF RELATED, MATRIX ASSOCIATED, ACTIN DEPENDENT REGULATOR OF CHROMATIN, SUBFAMILY A, MEMBER 2 | 1.9 | 1.6 | D |
| ZNF329 | ZINC FINGER PROTEIN 329 | 1.9 | 1.4 | D |
| RYBP | RING1 AND YY1 BINDING PROTEIN | 1.8 | 1.5 | D |
| TCF12 | TRANSCRIPTION FACTOR 12 | 1.8 | 1.3 | D |
| CTNND1 | CATENIN DELTA 1 | 1.7 | 1.4 | D |
| PBX3 | PRE-B-CELL LEUKEMIA TRANSCRIPTION FACTOR 3 | 1.7 | 1.5 | D |
| TFAP2A | TRANSCRIPTION FACTOR AP-2 ALPHA | 1.7 | 1.7 | D |
| SUV420H1 | SUPPRESSOR OF VARIEGATION 4-20 HOMOLOG 1 | 1.6 | 1.4 | D |
| ZNF148 | ZINC FINGER PROTEIN 148 | 1.6 | 1.3 | D |
| KLF13 | KRUPPEL-LIKE FACTOR 13 | 1.5 | 1.5 | D |
| SSBP3 | SINGLE STRANDED DNA BINDING PROTEIN 3 | 1.5 | 1.2 | D |
| SNAI2 | SNAIL HOMOLOG 2 | 1.5 | 1.2 | D |
| SERTAD2 | SERTA DOMAIN CONTAINING 2 | 1.4 | 1.4 | D |
| **Other** | | | | |
| DSC2 | DESMOCOLLIN 2 | 4.0 | 1.9 | U |
| TF | TRANSFERRIN | 2.2 | 2.0 | U |
| LNX1 | LIGAND OF NUMB-PROTEIN X 1 | 4.3 | 1.6 | D |
| INSIG2 | INSULIN INDUCED GENE 2 | 2.9 | 1.4 | D |
| IGFBP5 | INSULIN-LIKE GROWTH FACTOR BINDING PROTEIN 5 | 2.7 | 1.3 | D |
